# Study on the Main Influencing Factors in the Removal Process of Non-Stick Fluoropolymer Coatings Using Nd:YAG Laser

**DOI:** 10.3390/polym11010123

**Published:** 2019-01-12

**Authors:** Óscar Rodríguez-Alabanda, Pablo E. Romero, Carlos Soriano, Lorenzo Sevilla, Guillermo Guerrero-Vaca

**Affiliations:** 1 Department of Mechanical Engineering, University of Cordoba, Medina Azahara Avenue, 5, 14071 Cordoba, Spain; orodriguez@uco.es (Ó.R.-A.); guillermo.guerrero@uco.es (G.G.-V.); 2 IK4-Tekniker, Advanced Manufacturing Technologies Unit, Iñaki Goenaga Street, 5, 20600 Eibar, Spain; carlos.soriano@tekniker.es; 3 Department of Civil, Materials and Manufacturing Engineering, University of Malaga, Doctor Ortiz Ramos Street, 29071 Málaga, Spain; lsevilla@uma.es

**Keywords:** laser stripping, laser coating removal, FPE coatings, PTFE coatings, Nd:YAG laser

## Abstract

The coatings with fluoropolymer resins rich in fluorinated ethylene propylene (FEP) and polytetrafluoroethylene (PTFE) are applied as anti-adherent coatings on aluminum–magnesium substrates for use in food containers. In many cases, due to wear, they must be stripped for the application of a new coating on the same substrate. There are several processes for this: blasting, plasma, pyrolysis, chemical processes, laser, high pressure water, and combinations of these. This work focuses on the characterization of the main factors that condition the FEP coating removal process by a continuous wave (CW) Nd:YAG laser, and on the determination of the efficiency of this type of technology used for this purpose. Stripping surface per unit of time and energy consumption per unit area has been determined among other efficiency indicators. Regarding the characterization of the coating object of study, its thickness, surface roughness, contact angle, microhardness and absorbance-reflectance responses have been determined, and the results have been compared with those obtained in the case of PTFE. In addition, to evaluate the mechanical damage caused in the substrate after coating removal by (CW) Nd:YAG laser, the tensile strength, Vickers hardness, *R_a_* and *R_z_* roughness, and the substrate thickness have been measured and analyzed.

## 1. Introduction

Non-stick coatings based on fluoropolymers, such as polytetrafluoroethylene (PTFE), among others, can be applied to metals, enhancing their surface characteristics. Their application is extended over many industrial sectors: chemical [1], medical [2], automotive [3], textile [4], and food [4,5], among others. After use, these coatings degrade, wear out, and suffer deterioration [5]. Often, the cost of the piece does not justify its replacement by a new only due to the loss of properties in the coating layer, and a better solution is to perform the removal of the deteriorated coating [6] to apply a new one.

### 1.1. The Fluoropolymers

Conventional PTFE presents poor weldability, high melt viscosity, low radiation resistance, and high microvoid content. Is possible to improve its properties through the incorporation of bulky comonomers into the polymer main chain, optimizing the microvoid content, weldability, electrical insulation properties, and surface finishing, among others. However, this fluoropolymer has low tensile strength and wear resistance. These shortcomings pushed the experimentation to obtain novel tetrafluoroethylene derivatives by copolymerization with other type of monomers [6].

Fluorinated ethylene propylene (FEP) was the first copolymer obtained by copolymerization of TFE and hexafluoropropylene (HFP). This copolymer presents better impact strength, better wear resistance, and less permeability for organic solvents offering a resistance against chemical and weather agents, fire and radiation resistance, and electrical properties, similar to PTFE. Considering the specific application for cooking and bakery utensils, FEP improves the adhesion of the coating on the substrate and presents high thermal stability, although slightly less than PTFE [7].

These two different fluoropolymers and their combination shows extraordinary properties, summarized as an extreme chemical inertness [1], very low surface energy and, consequently, non-adhesion properties [8], excellent electrical insulation [9], high durability [10], and all this in a wide range of temperatures. Therefore, it could have great impact at an industrial level [6,11].

The ethylene propylene fluoride (FEP) is a fluoropolymer that allows for obtaining a continuous film on a substrate [12]. The coating is applied by spraying or wetting, for liquid resins, and by means of spraying or a fluidized bed for powder application, as described in detail in the previous works that were consulted [13]. Once the coating is applied, the pieces must be polymerized in an industrial oven between 350 and 380 ° C. The fluoropolymeric layers can reach thicknesses between 40 to 75 μm.

Polytetrafluoroethylene (PTFE) resins are usually applied with liquid formulations. Usually, the final layers polymerize by thermal action between 390 to 425 °C, and can reach thicknesses of 15 to 45 μm in the usual way, but PTFE finishes do not form a continuous layer, and their appearance is like the joint growth that takes place in a powder metallurgical sintering process.

### 1.2. Precedents and Objectives

Leaving laser ablation aside, many industrial techniques are used in the stripping of paints, among them, blasting [14,15], high-pressure water jets [16], the use of plasma [17], chemical procedures [18,19], cryogenic techniques [20], high-intensity light pulses [21], pyrolysis [22], and even a combination of them. However, there are only a few references about the research works in which they apply and study the stripping applications of fluoropolymer non-stick coatings.

The qualities of high chemical inertness, high surface hardness, high anchoring capacity on the substrate, and the property to remain unalterable even at relatively high temperatures, implies a great difficulty to properly remove this type of fluoropolymeric coating from the aluminum substrate.

It has been possible to consult a specific patent that has been registered for the stripping of fluoropolymers by Nd:YAG and CO_2_ laser technology [23]. This patent is limited to the description of the characteristics of the process without investigating energy efficiency, elimination rates, or the state of the substrate after the process.

The use of laser for this purpose has important advantages compared to other conventional techniques. Among them are the possibility of removing the fluoropolymeric layer with very little damage and even without any damage on the aluminum substrate [24], the possibility of selective stripping in multilayer coatings [25], the suppression of chemical products and the minimum production of polluting gases and vapors [26], and the high reproducibility of the process [27]. Therefore, the use of a laser beam for the removal of paints and other coatings, through the process known as laser ablation for stripping, has been of growing interest in recent decades.

There are some interesting studies cited in the literature on laser stripping on aluminum substrates, such as alloys EN AW 2024, EN AW 7075, and EN AW 5754. Advances have been developed in the aeronautical sector mainly with epoxy resins, epoxy polyamide, and polyurethane. Thus, excimer Nd:YAG and CO_2_ lasers [28], CO_2_ laser [29,30], diode laser [31], and Yb fiber laser [32] have been applied for various stripping jobs on these materials.

The way of driving the laser beam and the handling of CO_2_ lasers is complex in the practice, since the optical fiber system is not commercially available for this type of laser. Therefore, with the development of lasers that can be driven by high-power optical fiber, research on paint and coating stripping processes has received renewed attention. This is evident in various scientific investigations, culminating with the experimental and theoretical models recently published [33,34,35,36,37].

In this work, we will focus on the factors involved in the removal process of coatings made of FEP and PTFE by a continuous wave (CW) Nd:YAG laser. The objective of this study is to compare the stripping processes of FEP and PTFE non-stick fluoropolymer coatings, analyzing the qualities of each coating and its degree of influence in the energy efficiency and rate of stripping in addition to how the process affects the mechanical and superficial properties of the aluminum substrate.

## 2. Materials and Methods

### 2.1. Processing Procedure

The substrate used in the experiments is an Al–Mg alloy of type EN AW 5251 [38]. The advantages of this alloy include its good conformability, high mechanical qualities, low weight, and good behavior against thermal fatigue [39]. They are particularly suitable for use as a non-stick coating in the field of food. The thickness of the sheets used is 1.20 mm.

Eighteen samples of aluminum–magnesium alloy 140 × 120 × 1.2 mm^3^ were prepared: 8 units covered in PTFE, and another 8 units covered in FEP. Two units were left to study the substrate in the state of supply. With this number of test samples, all the measurements provided in the test plan have been carried out with total reliability. The chemical composition of EN AW 5251 H34 aluminum–magnesium alloy has been studied in an X-ray scanning electron microscope model JEOL JSM 6300, connected to an EDX unit, which allows displaying of the characteristic X-ray spectrum (Jeol USA Inc., Peabody, MA, USA). The obtained composition is shown in Table 1, comparable to that indicated in the supplier’s technical data sheet (CAMEBE, Castro Urdiales, Spain), according to UNE 38347:2004 [40].

**Figure 1:**
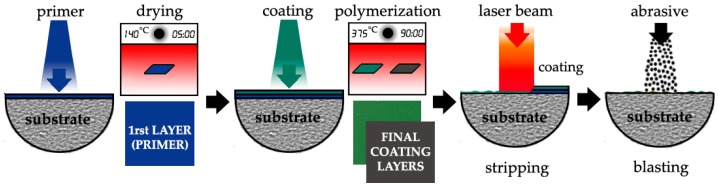
The processing cycle: priming + polymerization, final coating layers + polymerization, laser stripping and final cleaning by blasting; fluorinated ethylene propylene (FEP) (green) and polytetrafluoroethylene (PTFE) (dark).

The stripping conditions were evaluated and selected by a previous sensibility test. The state of the specimens submitted to the final experiment was evaluated after each cycle of coating application and stripping. The processing cycle is developed according to the recommendations of the specialist company in non-stick coatings, Tecnimacor (Villafranca de Córdoba, Spain), for both the coating of FEP and PTFE, as follows: (i) degreasing and light sandblasting of the substrate, (ii) application of the first coating layer (primer layer), (iii) application of the second layer and a third final layer of the coating and curing-polymerization in a static oven, (iv) stripping of the coating by laser, and (v) cleaning and preparation of the stripped surface by a blasting technique. This processing cycle was repeated 3 times for each type of coating. Figure 1 shows a diagram of the complete cycle described.

Prior to the multilayer coating of FEP, a liquid primer of Xylan 60/G4610 was applied and then dried at 120–150 °C for 5 min. On this first primer layer, a new layer of Xylan 1756/G3411 was applied. Finally, the powder coating Xylan FEP 80-550/G3131 was applied and the process finished with cure-polymerization in the oven.

Various industrial corporations, such as Whitford Ltd., Du Pont de Nemours & Co., Daikin, Ilag, or Grebe Group produce formulations for FEP and PTFE coatings. Concretely, the Whitford Company products [41] have been used as coatings in this work.

For the PTFE coating, a Marlite X-Treme primer was initially applied with drying at 120–150 °C for 5 min. Then, a layer of Marlite X-Treme 235.490 and, after, one more layer of Marlite X-Treme 245.491 before PTFE coating application and final curing in the oven.

**Figure 2:**
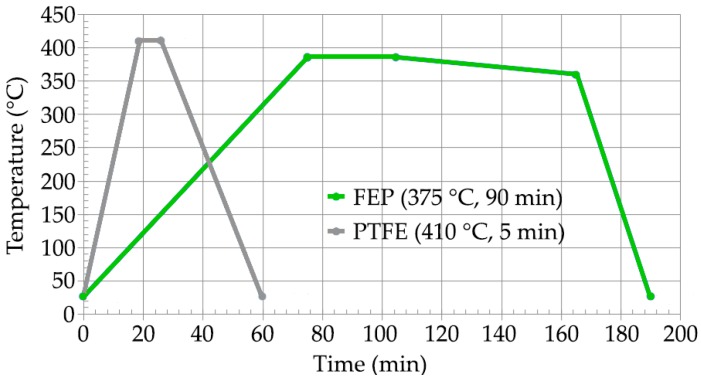
Curing-polymerization cycles for the FEP and PTFE formulations.

The thermal cycles applied in the case of FEP and PTFE have been carried out in in a Nabertehem NA 15/65 electric resistance static oven (Nabertehem GmbH, Lilienthal, Germany). This oven (Tmax = 650 °C, Pmax = 2700 W) is equipped with horizontal air circulation and is controlled by a programmer connected to a thermocouple in contact with the substrate. The polymerization cycles, shown in Figure 2, were performed according to the specifications of the manufacturer of the FEP and PTFE formulations (Whitford Ltd., Runcorn, UK) in terms of temperature and time.

### 2.2. Influence Factors in the (CW) Nd:YAG Laser Coating Removal Process

The characterization of the coatings, once polymerized and ready for use, was performed by measuring the thickness, surface rugosity, microhardness, static surface contact, backward and forward contact angles, and the determination of the laser light fraction reflected by the coating.

The final thickness of the coating was measured with a Fisher Dualscope MP0R series device based on the Foucault current method, according to the criteria of ISO 2808 [42]. This equipment allows to measure coating thicknesses non-destructively (Fischer Technology Inc., Windsor,CT, USA).

The Vickers microhardness was determined by the indentation procedure designed by Oliver and Pharr [37]. The Fischerscope H100 hardness measurement device (Fischer Technology Inc., Windsor, CT, USA) is a computer-controlled measuring system for microhardness testing and determination of material parameters according to ISO 14577 [43]. This device is automatized for applying and measuring, continuously and simultaneously, the indentation force and the penetration depth of the Vickers indenter mounted in its head, and is able to automatically select the exact load to penetrate 10% of the thickness of the surface measured, in this case, the coating layer.

Likewise, a wettability test was performed by a device equipped with an oscillating platform using the methodology proposed in previous works [44,45]. The tests have been performed with Pixelink CMOS Firewire monochrome camera, model PL-D795MU, equipped with a high magnification zoom objective (Navitar Company, Ottawa, Canada). All measurements were carried out in a controlled environment room at a temperature between 20 and 22 ° C, and relative humidity between 40% and 50%, applying Milli-Q water droplets with a volume of 100 μL.

**Figure 3:**
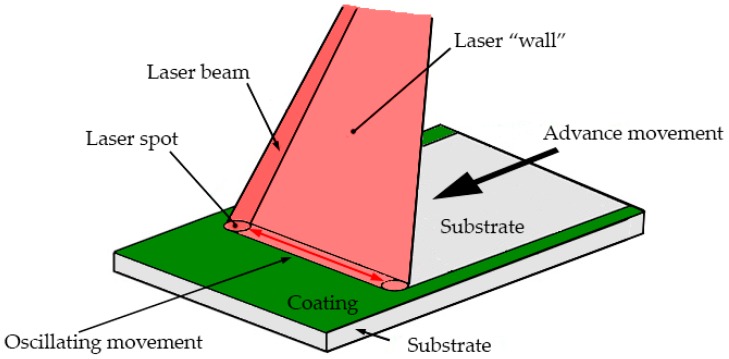
Laser working movements during coating removal process.

For the stripping, a continuous wave (CW) Nd:YAG laser Rofin-Baasel DY022 model was used (Rofin-Baasel España, S.L.Unip., Navarra, Spain) with a maximum power of 2200 W with λ = 1064 nm. The laser beam, which measures 1 mm in diameter, is focused on the area to be processed. The action of a converging lens device, that is equipped with a focal length of 200 mm (Raylase SS-LD-30 model, Raylase GmbH, Wessling, Germany), generates a laser “wall” that covers a working area of 120 × 120 mm^2^, as shown in Figure 3. The experience of the company, Tekniker (Tekniker Foundation, Eibar, Spain), has allowed for preselecting the intervals for the most appropriate power, scanning frequency, and speed values.

The laser absorbance spectra on the applied layers have been studied using a Bruker model FT-IR Tensor 27 device for infrared spectrometry (Bruker Biosciences Espanola S.A., Madrid, Spain). This test is used to characterize the applied fluoropolymer typology, allowing determination of a characteristic trace of the material to be studied through the absorbance level of the material with different wave numbers in the infrared field. On the other hand, the reflectance or the reflection capacity of the laser source on the coating was measured by the infrared spectrometer JASCO FT 4000 (Jasco Analítica Spain, Madrid, Spain).

After laser sensibility tests, high resolution images of the stripped surface where obtained by Leica DMV6 model digital microscope (Leica Microsistemas S.L.U., Hospitalet de Llobregat, Spain).

For the determination of the coating surface roughness, a series of measurements were made with a Mitutoyo portable device model Surftest SJ-201 (Unceta, Elgoibar, Spain) on each of the 140 × 120 mm^2^ specimens distributed homogeneously over the entire surface of each specimen. The ISO 4287 standard was applied (ISO, 1999) and a basic length of 0.8 mm and an evaluation length of 4 mm were selected for the determination of *R_a_* and *R_z_*, the mean and maximum roughness value, respectively. The variation of the thickness of the sheet was taken as an apparent average value through the reading in eight different points with a millesimal micrometer. To obtain the value of the thickness of the sheets used as substrate, a micrometer with millesimal appreciation was used. The readings per plate have been repeated 8 times/specimen, and the results correspond with the value of an apparent average. The surface roughness of the substrate was measured 5 times/specimen, perpendicular and transversal to the rolling direction of the sheet/substrate.

The mechanical properties of the aluminum substrate, before and after the repeated processing cycles, were determined using a Zwick Roell Z100 traction machine (Zwick Ibérica S.L., Barcelona, Spain), and the Vickers hardness was measured in a Zwick/Roell device ZHU250 TOP. The evolution of the mechanical properties of the aluminum substrate has been analyzed based on the results obtained after performing the corresponding tensile tests according to the standard UNE-EN ISO 6892-1:2010 [46]. The stress vs. % elongation curves and Vickers hardness have been determined as the average values, in a series of three tests, performed after each one of the removal/coating cycles with the aim of understand the evolution of these mechanical properties.

## 3. Results

### 3.1. Physical and Optical Properties of the Coatings

Once all the coating layers have been applied and polymerized on the substrates, both in the case of FEP and PTFE, the values of the parameters of surface roughness were measured just after each application cycle. The results of these surface roughness measurements are shown in Table 2.

**Figure 4:**
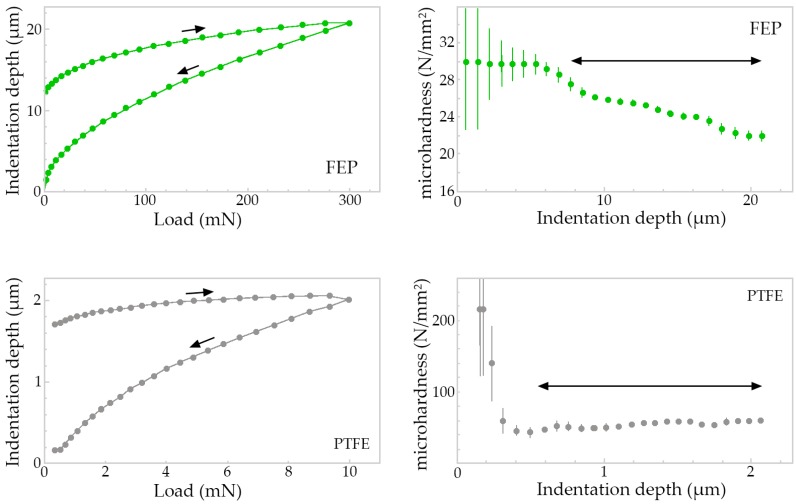
The load–unload curves (left) and microhardness (right) measured in FEP and PTFE coatings.

The microhardness of the deposited fluoropolymers was determined with the application of a load that reached 300 mN in the case of FEP and 10 mN in the case of PTFE. This load was gradually amplified in 25 stages and with a resting time between each two load levels of 1 s in both cases. It is important to indicate that the Vickers indenter always and automatically penetrates 10% of the thickness of the coating to be measured, hence, the scales and the evolution of the loading and unloading cycle are different for different indented materials. The results can be observed in the Figure 4, in which are reflected the load–unload curves (left) and microhardness (right) when the indenter achieves 10% of thickness on the specimens coated with FEP.

The microhardness values, expressed in N/mm^2^ or MPa, reach levels ranging from 28 to 38 N/mm^2^ at 8 μm depth to a value of 22 N/mm^2^ around 20 μm for the FEP fluoropolymer coating. In the case of PTFE fluoropolymer coating, hardness values ranging from 70 N/mm^2^ for a depth of 0.3 μm to 60 N/mm^2^ for a depth of 2 μm are observed.

It can be seen how the PTFE presents remarkably superior qualities in terms of the relationship between thickness and microhardness, reaching microhardness values more than two times higher than the FEP, for a thickness that is one third of that applied in the case of the FEP coating. It should be remarked that, in spite of the best properties measured in the case of PTFE, FEP coating allows for the accumulation of a greater thickness, thanks to its ability to melt layer by layer.

To obtain the wettability characteristics of both coatings, three drops of 100 μL demineralized water were deposited in different areas of each sample, by means of a manual micropipette. The sample rested on an inclined platform controlled by a motor, inclining at a step of 0.5 °/s. The data were evaluated through a digitalization of the image of the drop profile and adjusted to a specific theoretical profile. The values measured for the static, advance, and retreat contact angles in the FEP and PTFE samples are shown in Table 2.

**Figure 5:**
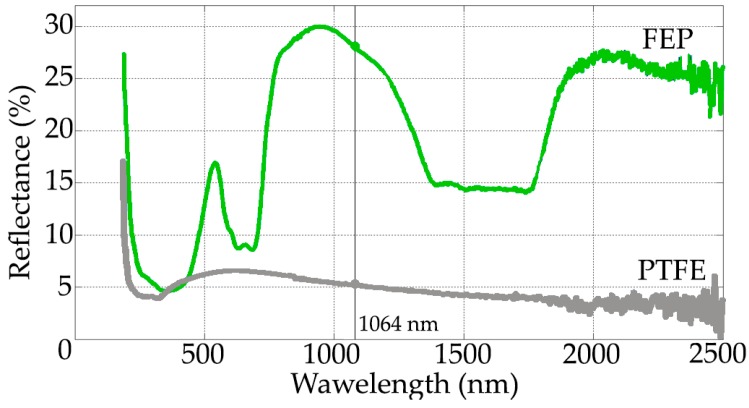
The reflectance (%) at 1064 nm presented in the FEP (up) and PTFE (down) coatings.

The reflectance graphs of the coating object of this study are shown in Figure 5. In this graph, it is observed that the fluoropolymer in dark color (PTFE), has a very low reflectivity at 1064 nm (laser emission wavelength), only 5%. Notwithstanding the fluoropolymer in clear green color (FEP) it reaches 27%–28% reflectivity. Observing the remarkable ability of the fluorinated ethylene propylene coating to reflect the laser source, it is intuited that the removal rate will be less efficient than in the case of PTFE fluoropolymer coating, which has a much lower reflectance at 1064 nm, as is shown in the Figure 5.

The values of coating thickness, microhardness, and contact angles measured in the non-stick coatings studied (see Table 3) agree with the recommendations of the supplier of these resins (Whitford Ltd., Runcorn, UK), and are comparable with those values consulted in the literature [1,45].

**Figure 6:**
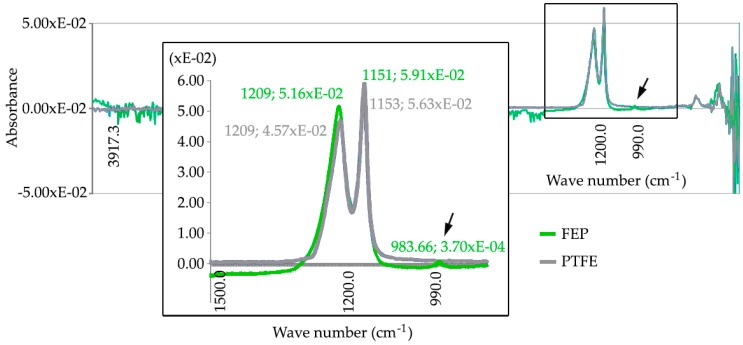
The characteristic absorbance (cm^−1^) presented in the FEP and PTFE coatings.

To characterize the applied coating, the absorbance level at different wave numbers in the infrared field has been determined. The wavelength was defined in cycles per centimeter (cm^−1^) and the characteristic absorbance curves for both FEP and PTFE coatings are shown in the Figure 6.

After taking several readings on the PTFE coating samples, two characteristic absorbance peaks have been detected at v = 1209 cm^−1^ and v = 1153 cm^−1^, while, in the case of FEP coating samples, characteristic absorbance peaks reached at v = 1209 cm^−1^, v = 1153 cm^−1^, and v = 983 cm^−1^. When an absorbance peak appears at the value 983 cm^−1^, the FEP can be perfectly identified by this specific characteristic that is not evidenced in the spectrum corresponding to PTFE [47,48].

### 3.2. Stripping of Non-Stick Coatings by (CW) Nd:YAG Laser

In order to find the most suitable values in each of the parameters, for a satisfactory removal of the coatings, a sensibility study was carried out. In this way, to sweep the surface to be treated with the continuous wave Nd:YAG laser device, a laser “wall” by high speed linear scanning of the laser spot has been generated. The linear scanning speed has been set at 5400 mm/s and the sweep width is 120 mm. The speed of the advancement movement oscillated between 2.5 and 10 mm/s. The delay or movement speed of the beam oscillation has been set at 200 μs.

The PTFE fluoropolymer test specimens have responded more efficiently to the removal than those of FEP. The initial sensibility test results of the FEP and PTFE are shown in Table 4.

**Figure 7:**
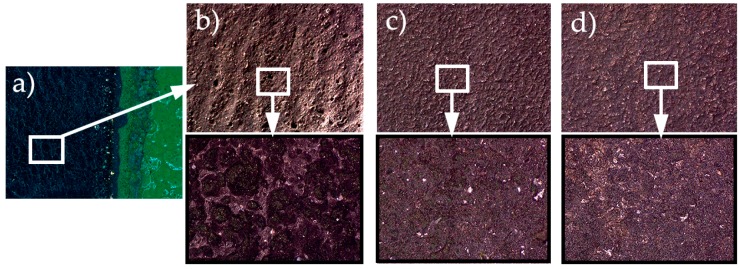
Surfaces after laser application: (**a**) magnifying glass test T6; (**b**) microscopy test T6, molten substrate; (**c**) microscopy test T8, clean substrate; (**d**) microscopy test T10, substrate with clear stains of coating rests.

On the other hand, as the process fluence (F) is defined as the energy supplied (E) per unit of stripped area (S), then the fluence was obtained as the quotient between the power of the laser (W) and the stripping rate (cm^2^/min). The most significant results of the final surface aspect in some of variants tested for the removal of the FEP are shown in Figure 7.

Focusing on the results obtained after FEP coating removal process, the conditions of the test T8 were selected for the final experiment. As seen in the microscope images in the Figure 7, the most aggressive tests (T6 and T7) cause the superficial melting of the substrate (see T6 in Figure 7b), while the less aggressive ones (T9 and T10) leave rests of the coating without completely removal (see T10 in Figure 7d). Test T8 has resulted in a clean, uniform surface with no coating residues.

With the selected parameter conditions, T8 for FEP coating and T3 in the case of PTFE, an energy density for the stripping of 160 J/cm^2^ was required for the FEP, and 50 J/cm^2^ coatings for the PTFE coatings. On the other hand, laser stripping rates of 150 cm^2^/min for FEP coating and 600 cm^2^/min for PTFE were required.

It should be noted that the plates that have been coated with PTFE show traces of cinder. Those coated with FEP have traces of polymeric material. After manual blasting and cleaning based on isopropanol, complete and clean removal is obtained in both cases.

As a pre-evaluation of a second option, the Q-Switching pulsed laser device (150W) was tested, removing the PTFE coating from a 9 cm^2^ surface specimen. Working at its maximum power, 4 passes were necessary to completely eliminate the coating, using 80 s, for this purpose. The removal rate obtained was 0.1125 cm^2^/s, a value up to 40 times lower than that obtained with the continuous wave laser device. The quality and surface cleanliness obtained in the substrate were superior to those obtained with the continuous wave laser, suppressing subsequent treatments. In addition, it is expected that this alternative technology minimizes the effect on the mechanical properties of the substrate by involving a much lower thermal input. However, an experimental study on this technique has not been developed with this device, and this second option was dismissed due to the low performance of the process compared to that obtained by (CW) Nd:YAG laser technology.

### 3.3. State of the Aluminum Substrate

The values of surface roughness and the variation of the thickness of the substrate are indicative of its state after each processing cycle. The evolution of these surface parameters is directly related with the superficial affection caused by the process. All the measures obtained are shown in Table 5.

**Figure 8:**
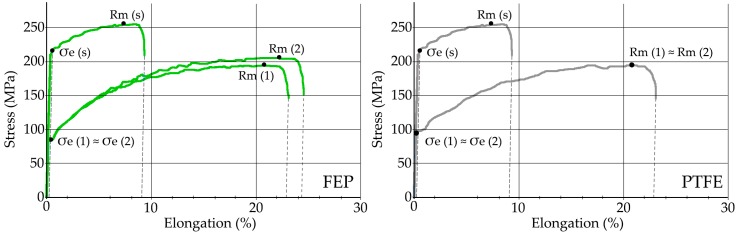
Stress–elongation (%) graphics of substrate coated by FEP and PTFE: (s) state of supply, (1) after removal-coating cycle 1, (2) after removal-coating cycle 2.

The evolution results corresponding to the mechanical properties of the aluminum substrate, after the successive removal/coating cycles, are represented in the stress vs. % elongation curves shown in Figure 8.

In the substrate in the state of supply, the value of elastic limit at 0.2% elongation σ_e_(s) = 217 MPa and break limit *R_m_*(*s*) = 256 MPa has been determined.

In the substrate coated with FEP, after a first processing cycle, the value of elastic limit decreased to σ_e_(1) = 86.5 MPa and break limit *R_m_*(1) = 195 MPa; after a second processing cycle, the values remained similar, without great variation, reaching σ_e_(2) = 86.1 MPa and *R_m_*(2) = 203 MPa, respectively. In the case of substrate coated with PTFE, after a first processing cycle, the value of elastic limit decreased to σ_e_(1) = 86.6 MPa and break limit *R_m_*(1) = 195 MPa; after a second processing cycle, the values remained similar, without great variation, reaching σ_e_(2) = 90 MPa and *R_m_*(2) = 203 MPa. It is intuited that these changes in the aluminum mechanical properties can be the consequence of the effect of “indirect annealing” caused by the polymerization process.

**Figure 9:**
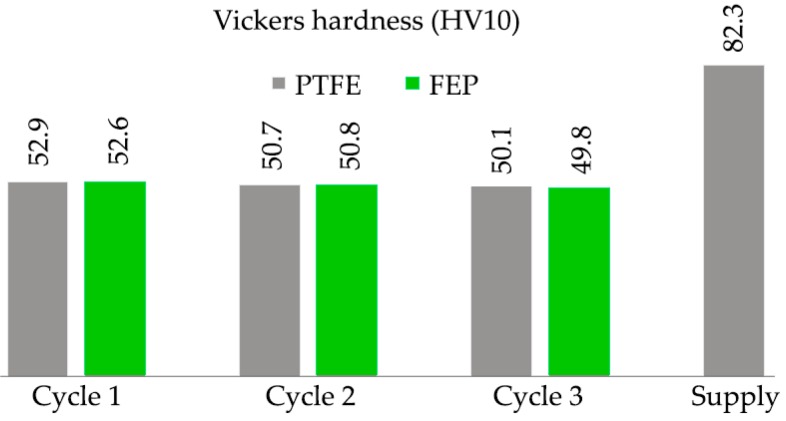
Vickers hardness values (HV10 method) of substrates EN AW 5251 after the thermal cycles of polymerization, coating, and stripping by laser, for FEP and PTFE coatings.

The results obtained from the Vickers hardness tests of the substrate, after each removal-coating cycle, are shown in Figure 9. Both in the substrate coated with FEP and in the substrate coated with PTFE, and once both coatings were removed by the (CW) Nd:YAG laser, the value of the Vickers hardness measured in the aluminum substrate shows an evident decrease, ranging from 82.3 HV10, in the supply state, to 49.8 HV10. This decrease in the Vickers hardness of the substrate is already evident after the first cycle of removal, and it is intuited that it is also a consequence of the effect of “indirect annealing” caused in the polymerization oven.

## 4. Discussion

### 4.1. Continuous Wave 1kW Nd:YAG Laser Efficiency

The laser equipment used has allowed for the elimination of FEP and PTFE coatings by a mechanism of thermal decomposition. The stripping rates obtained were 150 cm^2^/min for FEP and 600 cm^2^/min for PTFE. In other studies similar to this work, using laser sources for removal of epoxy polyester or polyurethane paint values between 50 and 140 cm^2^/min have been obtained [34,36,49]. The fluoropolymers appear to show a low stripping rate compared to other polymer coatings. In any case, the process of laser stripping of PTFE coatings presents more efficient values than in FEP coatings.

The yield rate for the FEP of 160 J/cm^2^ and the PTFE of 50 J/cm^2^ are consistent with studies performed for industrial paint stripping applications. For example, for the stripping of chlorine rubber paint and epoxy polyester paint, values are reached between 120 to 240 J/cm^2^ [33,49,50].

In this work, the average thickness of the PTFE coating is 19 μm, and that of the FEP is 61 μm, that is, the PTFE thicknesses are 3 times lower than those of the FEP. On the contrary, the microhardness shows values of the order of 2 times higher in the PTFE (60–70 MPa) than in the FEP (22–30 MPa). Finally, the reflectance at 1064 nm, which corresponds to the emission wavelength of the (CW) Nd:YAG laser, reaches 5% for PTFE and 27%–28% for FEP. In short, the higher stripping rate or, in other words, the best efficiency of the laser, is related to the lower coating thickness and with the lower reflectance value.

On the other hand, the microhardness of the coating seems to have a minor effect on the efficiency of the laser, since the softer polymer (FEP) has a worse stripping rate.

### 4.2. Substrate

The roughness values in the substrates after the stripping processes applied in this study have suffered slightly different trends, but not particularly significant [51]. It is observed that the roughness indicators Ra and Rz in the aluminum substrates, after the PTFE pickling, are slightly lower than after the pickling of the FEP. This is probably due to the greater difficulty of eliminating the FEP: two passes and a lower forward speed, 250 mm/min vs. 500 mm/min.

The mechanical properties of the substrate of EN AW 5251 undergo an important change. This modification is due to the thermal effect caused by the curing-polymerization (Figure 2) that produces the recrystallization of the structure [52]. The effect of laser stripping process on the mechanical properties of the substrate is of little relevance. A decrease in the tensile strength value *R_m_* is observed, which goes from 256 MPa in the state of supply to 196–205 MPa after curing the FEP or PTFE, that is, a loss of 27%–28%. After laser stripping, the variation is 195–203 MPa, a loss of between 1%–2%. The Vickers hardness shows similar behavior. In the state of supply, it reaches 82.3 HV10 and, after the polymerization, decreases to 53.5 HV10, a loss of 35%. After the laser stripping, the decrease in Vickers hardness in the substrate does not reach 1%.

Several published works demonstrate the impoverishment in the properties of substrates after laser stripping processes, including structural embrittlement due to hydrogen inclusions [53], or surface microfusion defects and a loss of fatigue properties [54]. Is noticeable that none of these effects seems to have been shown in the current study.

## 5. Conclusions

Analyzing the results in the present study, it is reasonable to reach the following conclusions:

- Stripping process by (CW) Nd:YAG laser is much more efficient for PTFE than for FEP coatings, after evaluating the process fluence (J/cm^2^) and the stripping rate (cm^2^/min).
- The greater efficiency of laser stripping technique is related to the lower reflectance of the fluoropolymer and the lower thickness value of the coating.
- The microhardness of the fluoropolymer coating does not show any relationship with the efficiency of the laser stripping.
- The Nd:YAG laser stripping of PTFE coatings seems to produce a smaller increase in *R_a_* and *R_z_* roughness on substrates than those produced in the case of FEP.
- The mechanical properties, tensile strength, and Vickers hardness of the aluminum alloy EN AW 5251 H34 suffer little significant variations (1%–2%) after successive cycles of (CW) Nd:YAG laser coating removal, both for PTFE and FEP.

## Figures and Tables

**Table 1:** Chemical composition (% by weight) of aluminum–magnesium substrate EN AW 5251 H34.

Elements	Si	Fe	Cu	Mn	Mg	Cr	Zn	Ti	Al
Analyzed	0.15	0.37	0.07	0.52	1.89	0.02	0.37	0.01	96.59
UNE 38347:2004	0–0.40	0–0.50	0–0.15	0.1–0.5	1.7–2.4	0–0.15	0–0.15	0–0.15	rest

**Table 2:** Surface roughness of the coatings after the application/polymerization of all layers.

Coating	Roughness	Cycle 1	Cycle 2	Cycle 3
FEP	*R_a_* (μm)	0.22	0.26	0.27
	*R_z_* (μm)	1.21	1.49	1.48
PTFE	*R_a_* (μm)	0.73	1.01	2.40
	*R_z_* (μm)	5.01	5.61	12.33

**Table 3:** Summary of physical properties on FEP and PTFE applied on substrates of EN AW 5251.

	Coating Thickness (µm ± σ)	Microhardness (MPa/µm)	Angle (°) Static/Advance/Retreat	Reflectance (%) at 1064 nm
**FEP**	61.3 ± 0.88	30/8	104/109/97	28–29
		22/20		
**PTFE**	19.09 ± 4.09	70/0.3	112/114/103	5–6
		60/2		

**Table 4:** Parameters selection tests for the Nd:YAG (CW) 2200W laser stripping of non-stick coatings (grey: final conditions selection for PTFE; green: final condition selection for FEP).

	Test Number	Power (W)	Scanning Frequency (Hz)	Advance Speed (mm/s)	Passes	Stripping Rate (cm^2^/min)	Process Fluence (J/cm^2^)
**PTFE**	T1	500	800	8.33	1	600	50
	T2	500	400	8.33	1	600	50
	T3	500	500	8.33	1	600	50
	T4	500	600	8.33	1	600	50
	T5	500	600	10	1	720	41.6
**FEP**	T6	800	200	2.5	2	90	266.6
	T7	900	200	2.5	2	90	300
	T8	800	200	4.16	2	150	160
	T9	600	200	5.83	2	210	85.7
	T10	600	200	9.16	2	330	54.5

**Table 5:** Roughness and thickness of the substrate after each coating removal/application cycle.

Coating	Property (Substrate)	State of Supply (Transversal/Longitudinal)	Cycle 1	Cycle 2	Cycle 3
FEP	*R_a_* (μm)	0.41/0.13	3.15	3.71	3.58
	*R_z_* (μm)	2.62/0.78	23.05	23.76	24.12
	Thickness (mm)	1.212	1.228	1.225	1.215
PTFE	*R_a_* (μm)	0.41/0.13	1.87	2.27	3.11
	*R_z_* (μm)	2.62/0.78	12.57	21.54	24.89
	Thickness (mm)	1.212	1.225	1.221	1.209
